# Dendritic Polyglycerolsulfate Near Infrared Fluorescent (NIRF) Dye Conjugate for Non-Invasively Monitoring of Inflammation in an Allergic Asthma Mouse Model

**DOI:** 10.1371/journal.pone.0057150

**Published:** 2013-02-21

**Authors:** Stefania Biffi, Simeone Dal Monego, Christian Dullin, Chiara Garrovo, Berislav Bosnjak, Kai Licha, Pia Welker, Michelle M. Epstein, Frauke Alves

**Affiliations:** 1 Cluster in Biomedicine (CBM scrl), Optical Imaging Laboratory, Trieste, Italy; 2 Institute for Maternal and Child Health, IRCCS Burlo Garofolo, Trieste, Italy; 3 Cluster in Biomedicine (CBM scrl), Bioinformatics, Trieste, Italy; 4 Department of Diagnostic Radiology, University Medical Center Göttingen, Göttingen, Germany; 5 Department of Dermatology, Division of Immunology, Allergy and Infectious Diseases, Experimental Allergy, Medical University of Vienna, Vienna, Austria; 6 Mivenion GmbH, Berlin, Germany; 7 Department of Hematology and Oncology, University Medical Center Göttingen, Germany; Klinikum rechts der Isar der TU München, Germany

## Abstract

**Background:**

Non-invasive *in vivo* imaging strategies are of high demand for longitudinal monitoring of inflammation during disease progression. In this study we present an imaging approach using near infrared fluorescence (NIRF) imaging in combination with a polyanionic macromolecular conjugate as a dedicated probe, known to target L- and P-selectin and C3/C5 complement factors.

**Methodology/Principal Findings:**

We investigated the suitability of dendritic polyglycerol sulfates (dPGS), conjugated with a hydrophilic version of the indocyanine green label with 6 sulfonate groups (6S-ICG) to monitor sites of inflammation using an experimental mouse model of allergic asthma. Accumulation of the NIRF-conjugated dPGS (dPGS-NIRF) in the inflamed lungs was analyzed *in* and *ex vivo* in comparison with the free NIRF dye using optical imaging. Commercially available smart probes activated by matrix metalloproteinase's (MMP) and cathepsins were used as a comparative control. The fluorescence intensity ratio between lung areas of asthmatic and healthy mice was four times higher for the dPGS in comparison to the free dye *in vivo* at four hrs post intravenous administration. No significant difference in fluorescence intensity between healthy and asthmatic mice was observed 24 hrs post injection for dPGS-NIRF. At this time point *ex-vivo* scans of asthmatic mice confirmed that the fluorescence within the lungs was reduced to approximately 30% of the intensity observed at 4 hrs post injection.

**Conclusions/Significance:**

Compared with smart-probes resulting in a high fluorescence level at 24 hrs post injection optical imaging with dPGS-NIRF conjugates is characterized by fast uptake of the probe at inflammatory sites and represents a novel approach to monitor lung inflammation as demonstrated in mice with allergic asthma.

## Introduction

Currently, NIRF imaging is a common technology in preclinical studies that obtains functional information *in vivo* over time for assessment of antibody binding, protein expression, enzyme activities, cell tracking etc. [1]–[3]. Optical imaging provides relatively inexpensive and non-harmful methods and is preferred over other imaging methods used in preclinical research and drug development, such as PET and SPECT that are more complex to perform. However, the penetration depth of typically up to 4 cm in the near infrared range (NIR) limits its clinical application to endoscopic techniques and structures beneath the skin or fluorescence guided surgery [4]. Crucial for the success of *in vivo* NIRF imaging will be the development of dedicated NIRF probes for distinct targets of molecular events characterizing different diseases. So far, these probes, based on their mechanisms of target-detection can be divided into four groups: passive probes to image areas with increased blood supply [5], target-specific fluorescent probes which are directed against molecular and/or disease-specific markers [6], fluorescent labels to track injected fluorescence stained cells [7], and application of smart probes activated by enzymes for the detection of molecular events [8].

NIRF imaging in lung disease models has remained challenging due to the high scattering nature of the lung and its comparable deep location. Recent application of novel non-invasive imaging technologies in mouse models of asthma has enabled functional and longitudinal *in vivo* monitoring of disease, validation of novel biomarkers, and direct tracking of immune cells within tissues. Novel methods for *in vivo* monitoring of lung inflammation in mice include the utilization of smart-probes activated by MMPs or cathepsin, enzymes known to be involved in lung inflammation [9]–[11].

Airway inflammation is a central component of asthma that consists of edema, cellular infiltration, particularly of eosinophils, neutrophils, activated T lymphocytes and mast cells, increased airway secretions, and deposition of excess collagen. Therefore mouse models of asthma present attractive tools for evaluating probes suitable for *in vivo* molecular imaging of lung inflammation [12], [13].

**Figure 1:**
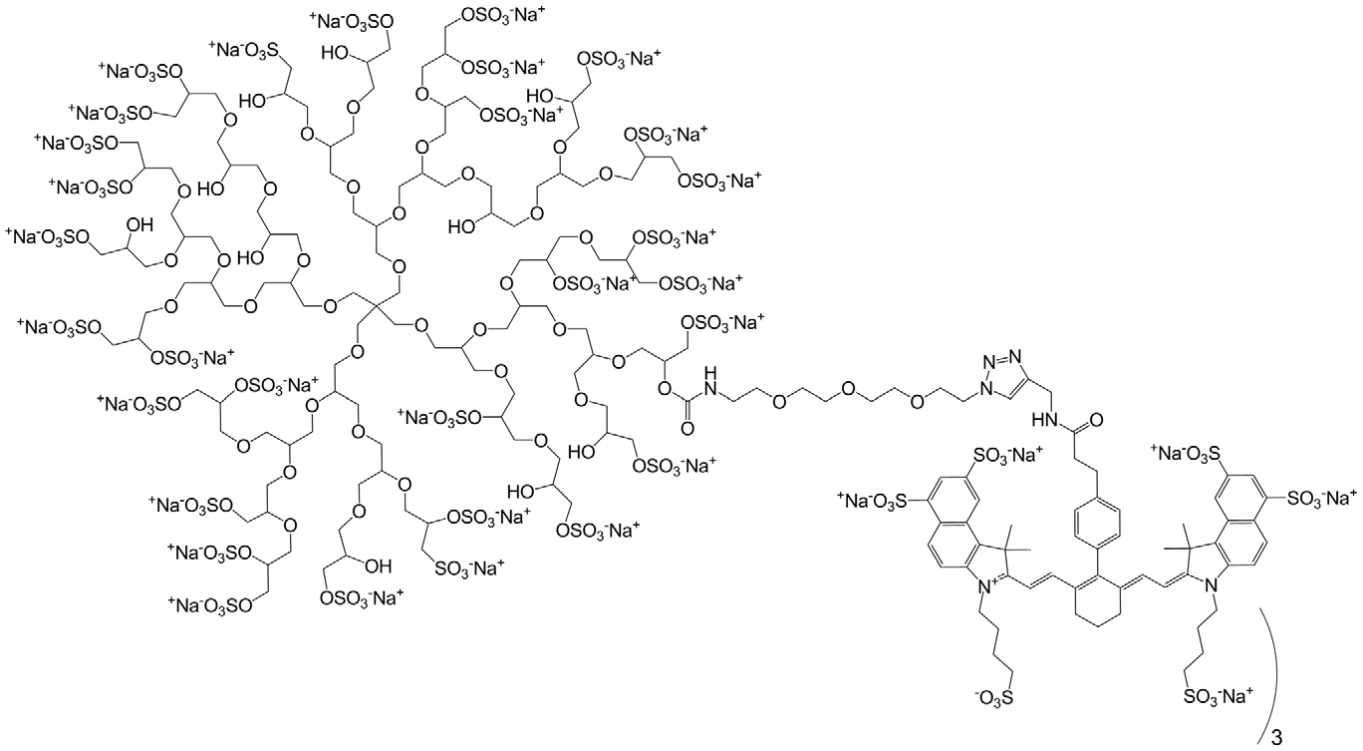
Chemical structure of dPGS-NIRF. The chemical structure indicates the linker structure and connection to the dye (approx. 3 dyes per polymer). Please note that the polymer is not depicted in original molecular weight, but is shown only as principle sketch.

Using a model of allergen-induced lung inflammation, we applied fluorescence imaging in combination with near-infrared (NIR) fluorescently-labeled dendritic polyglycerol sulfates (dPGS), a class of compounds that selectively bind to mediators of inflammatory processes such as L- and P-selectin and C3/C5 complement factors [14], [15]. The role of selectin-ligand interactions in allergic asthma is well established, making them an attractive target for visualization of inflammation [16]–[19]. For example, reduced airway hyperresponsiveness in asthma in L-Selectin-deficient mice has been reported [19]. Furthermore, studies show that dPGS is transported into inflammatory cells e.g. in activated mononuclear cells [20], [21]. Generally, dPGS consists of a highly branched (dendritic) polyglycerol core, which due to the large amount of hydroxyl end groups enables high functionalization. In our case, sulfate groups were generated from the hydroxyl groups, thereby creating the highly charged, polyanionic dPGS compound (Figure 1). dPGS acts via a multivalent binding mechanism mimicking naturally occurring selectin ligands [20], with a clearly demonstrated dependence of the binding affinity from molecular weight and degree of sulfation [15], [21]. Sulfation of the hydroxyl groups in the polymer established a multivalent polyanionic entity with high affinity for L- and P-selectin [22]. Anti-inflammatory property of dPGS in much higher concentrations has been reported to occur as a result of a multivalent interaction enabled by the multitude of sulfate groups. For instance, binding of dPGS to L-selectin on leukocytes and P-selectin on inflamed vascular endothelium reduces leukocyte extravasation by shielding the adhesion molecule [22]. Additionally, inhibition of C5a generation inhibits leukocyte chemotaxis [14], [22].

The compound used herein has a core molecular weight of about 6000 Da, imparting high binding affinity of the respective polysulfate [21] and at the same time having a reasonable molecular weight range for sufficient distribution and excretion [20], as well as chemical derivatization in order to conjugate NIRF dyes to the polymeric entity. The aim of the present study was to assess the capacity of dPGS conjugated with a near infrared fluorescent (NIRF) dye related to indocyanine green (dPGS-NIRF) to detect inflammatory sites in lungs by NIRF optical imaging analysis in a mouse model of asthma and to compare dPGS-NIRF to the commercially available smart-probes MMPSense and ProSense.

## Results

### OVA-immunization and challenge-induced allergic inflammation and extensive mucus hypersecretion in the lungs, and elevated serum OVA-specific IgG1

**Figure 2:**
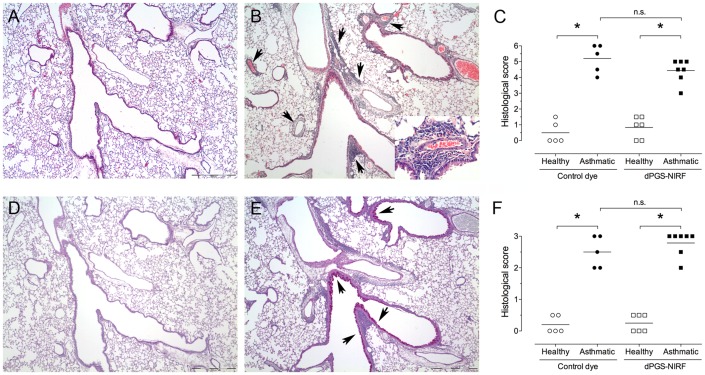
Allergic inflammation and mucus hypersecretion in the lungs of asthmatic, but not control mice. Lungs were harvested 76 hrs after the final ovalbumin (OVA) challenge meaning 4 hrs post i.v. probe injection. Representative H&E (A and B) and PAS (D and E) stained photomicrographs of lungs from healthy (A and D) or asthmatic mice (B and E) are shown (magnification 40×, inset 400×). (B) Arrows indicate inflammation, and in (E) arrows indicate mucus hypersecretion. Allergic inflammation (C) and mucus hypersecretion (F) scores in H&E and PAS stained lung sections, respectively, of healthy (open symbols) or asthmatic mice (filled symbols). Each symbol represents individual mice (n = 5–6 for healthy groups and n = 5–7 for asthmatic groups), and line represents group mean. One-way ANOVA followed by Tukey's multiple comparison test (*P<0.05) was used to compare differences between groups.

Allergic asthma inflammation and mucus hypersecretion in mice was induced by two intraperitoneal injections and subsequent intranasal challenges with OVA. Figure 2 illustrates lung histology from H&E and PAS-stained lung sections of asthmatic and healthy control mice. H&E staining revealed that no inflammatory infiltrates were present in lungs from healthy mice (Figure 2A). In contrast, immunized mice had dense inflammatory infiltrates containing predominantly eosinophils, as well as macrophages and lymphocytes surrounding blood vessels, and large and small airways (Figure 2B). The extent of allergic inflammation was evaluated by assessing the total surface area and location of leukocyte infiltration in lung sections (Figure 2C). Mice with allergic inflammation have histological scores of 5.2±0.4 (dPGS-NIRF group) and 4.4±0.3 (dye group) compared to healthy controls with 0.5±0.3 (dPGS-NIRF group) to 0.8±0.3 (dye group), demonstrating that diseased mice have lung inflammation affecting more than two thirds of the examined lung sections with infiltrates present in the hilum extending to the lung periphery.

To assess mucus hypersecretion, adjacent lungs sections were stained with PAS. As expected, only rare mucus producing cells were detected in the central airways of healthy control mice (Figure 2D), whereas numerous mucus producing cells were observed in asthmatic mice (Figure 2E). Histological evaluation revealed that asthmatic mice have histological scores for mucus overproduction of 2.8±0.4 (dPGS-NIRF group) and 2.5±0.5 (dye group) compared to healthy controls with 0.3±0.3 (dPGS-NIRF group) to 0.2±0.3 (dye group) (Figure 2F), indicating that mucus hypersecretion extended to the periphery of the diseased lungs.

We also tested serum OVA-specific Th2-isotype antibody titres. While no OVA-specific antibodies in sera were detected before immunization with OVA, high titres (≥1∶7812500) of OVA-specific IgG1 were detected in all OVA-sensitizated and challenged mice (results not shown), further supporting presence of allergic immune responses in both investigated groups.

### Allergic asthma can be successfully visualized by combination of dPGS-NIRF probe and *in vivo* optical imaging

To visualize allergic inflammation *in vivo*, we injected dPGS-NIRF and the control dye i.v. into the tail vein at 72 hrs after last OVA challenge, when we expected that allergic inflammation in the lung is at its peak. Asthmatic and healthy mice were imaged at 4 and 24 hrs post dPGS-NIRF or unconjugated NIRF dye injection as control.

**Figure 3:**
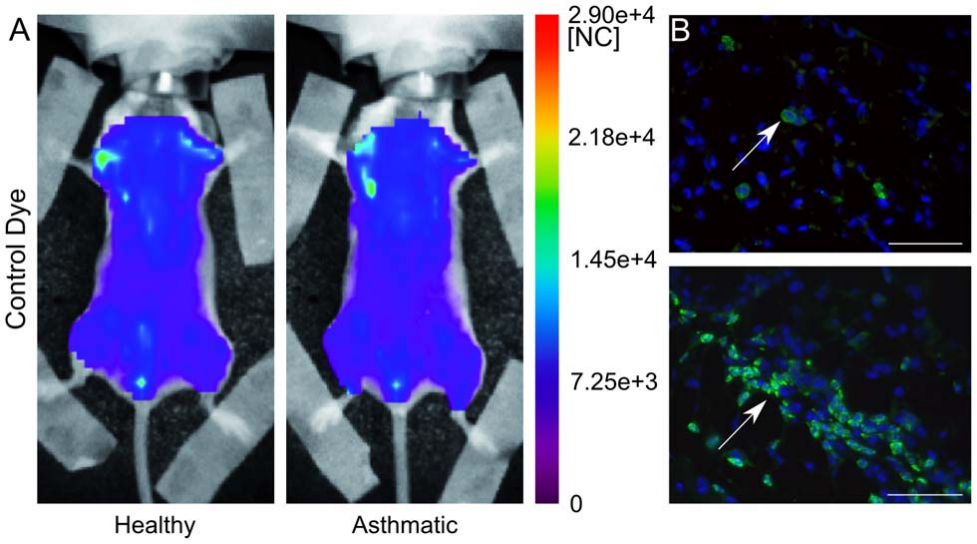
In vivo distribution of free dye (indocyanine green) 4 hours post probe injection and 76 hours post last OVA challenge. Panel A: whole body fluorescence intensity distribution of a representative healthy and asthmatic mouse displayed in normalized counts [NC]. Panel B: Fluorescence microscopy images of F4/80 stained macrophages and DAPI stained cell nuclei of lungs isolated from asthmatic and healthy mice injected with the NIRF labeled control dye and sacrificed 4 hrs post injection demonstrate no fluorescent control dye. F4/80 expression on macrophages are depicted in green, cell nucleus in blue, control dye was not detected (bar = 50 µm). In the healthy model few macrophages have been detected with respect to the asthmatic mouse, where cluster of cells are visible (see white arrows indicating macrophages). In both samples no unconjugated NIRF dye 6S-ICG has been visualized.

**Figure 4:**
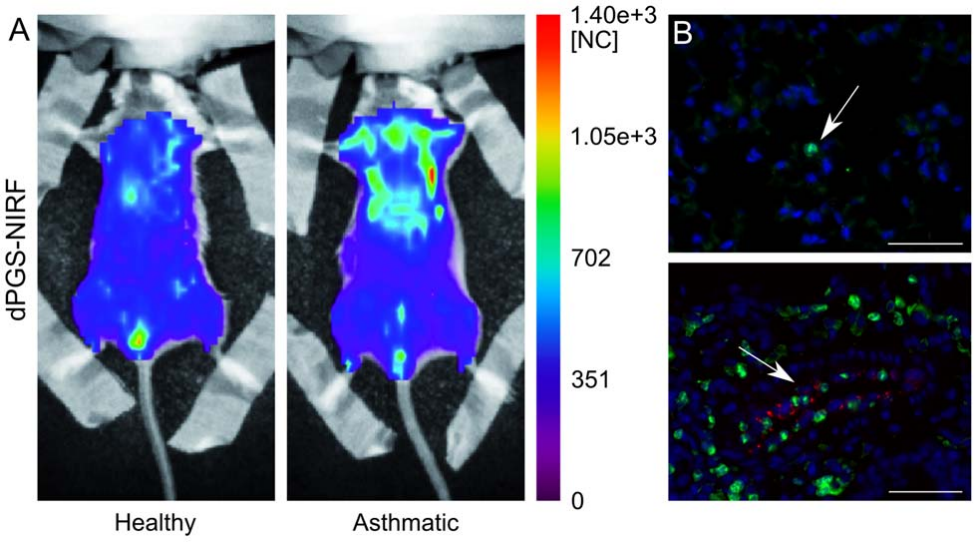
In vivo distribution of dPGS-NIRF 4 hours post probe injection and 76 post last OVA challenge. Panel A: whole body fluorescence intensity distribution in a representative healthy and asthmatic mouse displayed in normalized counts [NC]. Stronger fluorescence intensity over the lung area of the asthmatic mouse can be seen. Panel B Fluorescence microscopy images of F4/80 stained macrophages and DAPI stained cell nuclei of lungs isolated from asthmatic and healthy mice injected with dPGS-NIRF and sacrificed 4 hrs post injection. F4/80 expression on macrophages are depicted in green, cell nucleus in blue, dPGS-NIRF displayed in red (bar = 50 µm). In the healthy model, few macrophages and no probe localization have been detected. In the asthmatic mouse, cluster of macrophages are detectable (see white arrows) and the dPGS-NIRF probe was visualized in the same region of macrophages.

Figures 3 and 4 illustrate the distribution of the control dye and dPGS-NIRF, respectively, after 4 hrs in the thoracic area of asthmatic in comparison to healthy mice. A slight increase of fluorescent signal was recorded after injection of control dye in asthmatic mice in comparison to healthy mice (Figure 3A). In order to localize the dPGS-NIRF probe within inflamed lung region we applied fluorescence microscopy in combination with immunofluorescence staining of macrophages by the use of an antibody against F4/80, a 160 kDa cell surface glycoprotein that is widely expressed on mature tissue macrophages. As shown in Figure 3B a higher amount of macrophages was clearly detectable in lungs of asthmatic mice in comparison to healthy controls. The Control dye was not detected in lung sections of asthmatic mice using fluorescence microscopy (Figure 3B). In contrast, higher fluorescence intensity was detected in the thoracic region of asthmatic mice 4 hrs post dPGS-NIRF probe injection (Figure 4A). Moreover, fluorescence microscopy of lung sections of asthmatic mice confirmed dPGS-NIRF probe localization in areas where F4/80 stained macrophages could be detected, which demonstrated that dPGS-NIRF accumulates especially in the inflamed region of lungs of the pathological model (Figure 4B).

**Figure 5:**
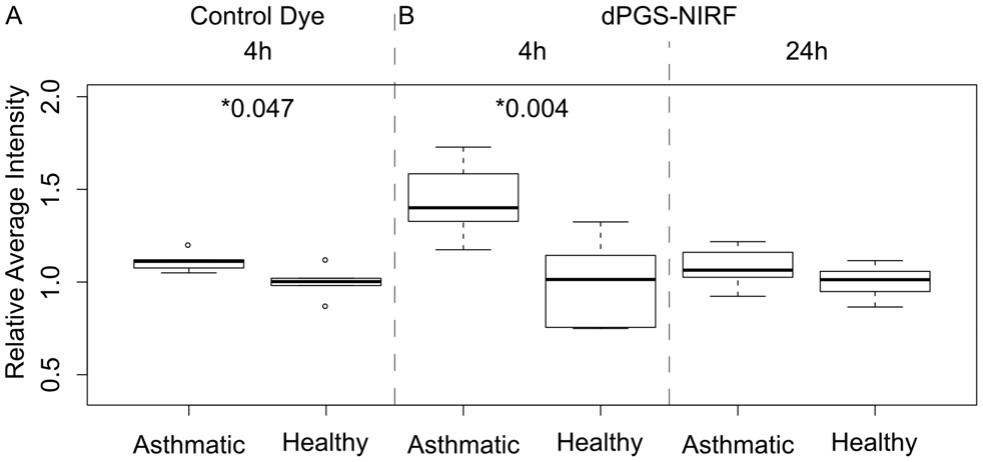
Quantification of in vivo imaging results of dPGS-NIRF and pure dye. Box plots of ratios of average fluorescence intensity over the lung area compared with the mean value of each control group respectively are reported for asthmatic and healthy mice. Mice treated with free dye 4 hrs post injection showed a slight increase in fluorescence signal in asthmatic mice (n = 5) when compared to healthy mice (n = 5; increase in average ∼11%, p-value = 0.047, panel A). Mice treated with dPGS-NIRF probe 4 hrs post injection (healthy n = 6, asthmatic n = 6) showed an increased fluorescence signal in the thorax in asthmatic mice (increase in average ∼44% with p-value = 0.004, panel B left side). At 24 hrs post injection fluorescence signals over the lung areas of healthy (n = 5) and asthmatic mice (n = 10) shown no difference (difference ∼8%, p-value = 0.162, panel B right side). Both control dye and dPGS-NIRF probe were injected 72 hrs after last aerosol challenge. Note, intensity ratios were used to compare probes with different brightness, therefore the box plots are depicted in the same scale.

Fluorescence signals obtained with *in vivo* imaging were quantified and intensity ratios were calculated as described in the Material and Methods. As depicted in Figure 5A, at 4 hrs post injection of control dye, we observed a slight increase in fluorescence signal in asthmatic mice when compared to healthy mice (increase in average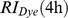 ∼11%, p-value = 0.047), most probably due to an increase in the vascular flow in the inflamed lungs. In contrast, dPGS-NIRF increased the fluorescence signal in the thorax of asthmatic mice dramatically, as seen by an average 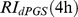 ∼44% with p-value = 0.004. Moreover, a direct comparison of the contrast (RI) between dPGS-NIRF and free dye in the asthmatic mice revealed a 30% higher 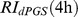 than 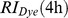 (p-value = 0.005) at this time point. At 24 hrs post dPGS-NIRF injection, fluorescence signals over the lung areas of healthy and asthmatic mice were not longer distinguishable (average 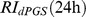 difference ∼8%, p-value = 0.162) (Figure 5B). *In vitro* analysis of serum binding of ICG as well as of 6S-ICG demonstrate that ICG completely binds to serum proteins (23), whereas less than 40% of 6S-ICG was bound to serum proteins (data not shown).

### Ex vivo optical imaging confirmed the *in vivo* results

**Figure 6:**
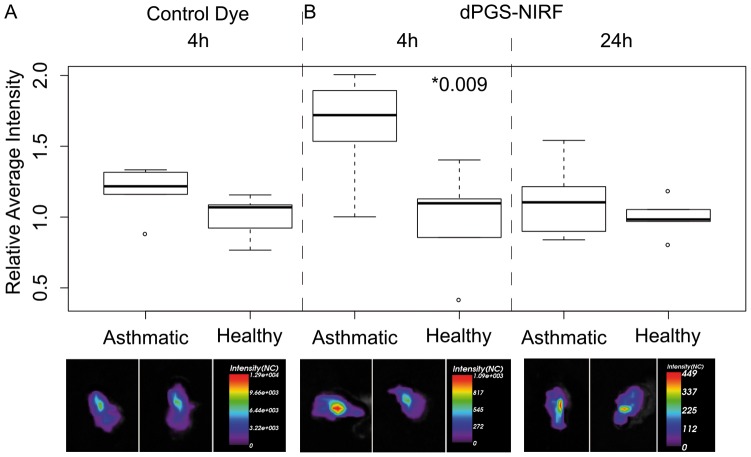
Ex vivo imaging results of dPGS-NIRF and pure dye. Box plots of ratios of average fluorescence intensity over the explanted lungs compared with the mean value of each control group respectively are reported for asthmatic and healthy mice treated with free dye 4 hrs post injection (panel A), and treated with dPGS-NIRF probe 4 hrs (panel B left side) and 24 hrs (panel B right side) post injection. The corresponding fluorescence intensity images of representative lungs are given at the bottom of each box plot. A significant difference between the fluorescence intensity within the lungs of asthmatic (n = 5) and healthy mice (n = 5) was observed 4 hrs post injection of the dPGS-NIRF conjugate (difference of ∼65%, p-value = 0.009), but not of the control dye (healthy n = 5, asthmatic n = 5; difference of ∼18%, p-value = 0.127). Both control dye and dPGS-NIRF probe were injected 72 hrs after last aerosol challenge. Note, intensity ratios were used to compare probes with different brightness, therefore the box plots are depicted in the same scale.

To confirm the *in vivo* imaging findings immediately after the last imaging, we imaged the lungs ex-vivo using an Optix MX2 system. Ex-vivo imaging avoids autofluorescence of other organs and absorption and scattering within the body and fur. This increases both specificity and sensitivity of probe detection. In accordance to the *in vivo* results, we found a significant difference between the fluorescence intensity within the lungs of asthmatic and healthy mice 4 hrs post injection of the dPGS-NIRF conjugate (difference of 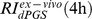 ∼65%, p-value = 0.009), but not control dye (difference of 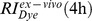 ∼18%, p-value = 0.127) (Figure 6A and 6B). At 24 hrs post administration of dPGS-NIRF, the observed fluorescence intensity over the lungs was reduced to about 30% of the intensity measured 4 hrs post injection. Moreover, the difference in fluorescence intensity between healthy and asthmatic mice dropped down to ∼10% and was not significant (difference of 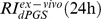 ∼10%, p-value = 0.323) (Figure 6B).

### Comparison of dPGS-NIRF with commercially available smart-probes

Commercially available smart-probes ProSense and MMPSense, activated by cathepsins and MMPs, respectively, were used for imaging lung inflammation [9]. Both smart-probes were injected at 72 hrs after the last OVA challenge and imaged after 24 hrs, according to probe manufacturer's recommendations. The intensity difference from the thoracic region between healthy and asthmatic mice was ∼27% (p-value = 0.013) after administration of ProSense and ∼83% after injection of MMPSense but with no statistical significance (p-value = 0.093) (Table 1).

**Table 1:** Calculated average fluorescence intensity ratios 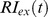 between healthy and asthmatic mice after injection of control dye, dPGS-NIRF, or two commercially available probes: ProSense and MMPSense.

	Control Dye	dPGS-NIRF		ProSense	MMPSense
measurement time (hours)	4	4	24	24	24
*in vivo*	1.11±0.06 (0.047)	1.45±0.20 (0.004)	1.08±0.10 (0.162)	1.27±0.02 (0.013)	1.83±0.50 (0.093)
*ex vivo*	1.18±0.18 (0.127)	1.65±0.35 (0.009)	1.10±0.22 (0.323)	n.d.	n.d.

Results are shown as mean calculated average fluorescence intensity ratios ± standard deviation, while statistical significance between each pair of control and asthmatic mice is given by p-value for the Welch-T-Test in brackets. Legend: n.d. – not done.

## Discussion

In this study, we present a novel approach for functional *in-vivo* imaging utilizing a dendritic polyglycerolsulfate conjugated to a NIRF dye related to ICG (dPGS-NIRF) in combination with optical imaging to monitor sites of inflammation in the lung by applying an experimental model of allergic asthma [12].

We successfully demonstrated that the applied dPGS-NIRF probe accumulates to inflammatory sites within the lung already 4 hrs after probe administration. The results show a significant four times stronger contrast of the fluorescence intensity of the dPGS-NIRF probe compared to the free dye in lungs of asthmatic in comparison to healthy mice. At this time point fluorescence microscopy confirmed the localization of the dPGS-NIRF probe within the inflamed lungs in areas where F4/80 stained macrophages could be detected and histology demonstrated the presence of inflammatory infiltrates in more than two thirds of the examined lung sections. Therefore, dPGS-NIRF is suitable to monitor inflamed lungs by NIRF imaging.

Furthermore, at 4 hrs post injection, the calculated 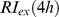 was significantly lower in asthmatic lungs after administration of free dye. These results indicate that a specific target in the model appears to be involved. dPGS-NIRF exerts high-affinity binding to positively charged protein motifs e.g. P- and L-selectin as well as to C3/C5 complement factors [22]. The selectivity is demonstrated by the very low affinity for E-selectin compared to P and L-selectin, which dPGS bind to with nanomolar affinity *in vitro* [20], [22]. Furthermore, dPGS accumulates in inflamed tissue by a not yet understood cellular uptake mechanism into macrophages and endothelial cells, but not into lymphocytes. This was shown for example by fluorescence microscopy of liver tissue specimens after dPGS-NIRF application that depicted accumulation in rat liver macrophages (Kupffer cells) and of A549 tumor cells as well as of activated, LPS-stimulated mononuclear cells, both demonstrating accumulation of dPGS [22].

The underlying chemical structure of the polymer in published studies [22] is based on a polyglycerol core of 6000 Dal, whereas different dyes were attached to the polymer, such as a visible cyanine dye or a NIRF dye in a ratio of approx. 1 dye per polymer, yielding identical selectin-binding properties. The conjugate used herein employs the same polymer, but a more hydrophilic indocyanine dye with 6 sulfonate groups (6S-ICG) added to the fluorophore structure. Coupling to the dPGS could be achieved at a dye-to-polymer ratio of 3 without signs of aggregation known to be induced by more lipophilic indocyanine dyes in bioconjugates, as described in [20].

Previously, studies show also inflammation-specific imaging with dPGS-NIRF in an animal model of collagen induced rheumatoid arthritis using the preceding conjugate with a lipophilic indocyanine green label. Comparable to our study, the authors demonstrated a fast and selective uptake of the probe with a 3.5 fold higher fluorescence difference between healthy and diseased joints and a signal peak at 1 hr after probe administration. Together with a rough estimation of a blood half-life of shorter than 1 hr by employing the eye fluorescence as a provisional solution to monitor blood kinetics, they postulate targeting mechanisms not yet fully understood [20] whereby dPGS-NIRF binds to mediators of inflammation.

Interestingly, the high contrast between the fluorescence intensity of dPGS-NIRF in the asthmatic and healthy groups was not observed after 24 hrs. This might be explained in part by shedding of P- and L-selectins from the cell surface after binding of dPGS-NIRF [23]. Bound dPGS-NIRF probes will be removed from the cells resulting in the reduction of fluorescence intensity to background after 24 hrs.

The MMPSense and ProSense probes, which are activated in the presence of inflammation-associated enzymes such as cathepsin and MMP's that are present in the lungs during allergen challenge are successfully used by others, for example to detect lung inflammation and rapidly screen for new drug effects [9]–[11] as well as to visualize colon adenomas [8]. Similar to our study, Cortez-Retamozo et al. demonstrated fluorescence differences between asthmatic lungs and healthy controls by applying the same amount of MMPSense or ProSense however by using fiberoptic bronchoscopy and fluorescence molecular tomography (FMT) [9]. Others also reported that the *in vivo* profile of cysteine protease activation was depicted by FMT in a mouse model of acute airway inflammation by LPS-induction [10], [11].

These smart probes exhibit slower kinetics due to their activation mechanism, demonstrating maximal fluorescence intensity within the lungs about 24 hrs after probe injection [9]. The application of these enzymatically activated probes is often hampered by the fact that despite a dramatic increase of their fluorescence intensity over inflammatory areas, the activated probes do not remain at the site of interest for very long and instead produce a strong liver signal due to their excretion pathway.

In conclusion, we present a novel *in vivo* NIRF imaging probe for detection of inflammatory reactions within the lungs of mice, as demonstrated in mice with allergic inflammation, by utilizing a dendritic polyglycerolsulfate NIRF dye conjugate known to bind to selectins and complement factors. The greater fluorescence intensity of dPGS-NIRF in inflammation of mice with allergic asthma in combination with rapid kinetics makes dPGS-NIRF a powerful probe candidate to monitor inflammation processes and responses to therapy in experimental mouse models of lung disease.

## Materials and Methods

### Mice

Female BALB/c mice (4- to 6-weeks old) were purchased from Charles River and maintained with ad libitum food and water. All the experimental procedures were performed in compliance with the guidelines of European (86/609/EEC) and Italian (D.L.116/92) as well as German laws and were approved by the Italian Ministry of University and Research and the Administration of the University Animal Facility, Trieste, as well as by the administration of Lower Saxony, Germany.

### Synthesis of a dendritic polyglycerol sulfates NIR dye conjugate probe (dPGS-NIRF)

dPGS was synthesized by anionic polymerization of glycidol and subsequent sulfation using SO3/pyridinum complex according to Türk and colleagues [14]. Conjugation of dPGS to an NIRF dye (based on indocyanine green chromophore; derivative with reactive group for conjugation) are described [20] elsewhere. Briefly, the polyglycerol intermediate was reacted with an aliphatic linker chain followed by the sulfation reaction. To this linker, a novel NIRF dye (6S-ICG propargyl; mivenion GmbH) was conjugated followed by high-performance liquid chromatography (HPLC) purification yielding dPGS-NIRF with a mean dye-to-polymer ratio of 3 and an average molecular weight of 19000 Da. The degree of sulfonation was 85% (elementary analysis) and the polydispersity index (PDI) within 1.6–1.8 (measured for the polyglycerol intermediate using GPC). The dye used herein is a hydrophilic version of the previously described indocyanine green label, with 4 additional sulfonate groups in the molecule resulting in a 6-fold sulfonated entity of maximal hydrophilicity for this type of NIR fluorophore. The chemical structure is depicted in Figure 1. Absorption maxima in PBS were 710 and 795 nm, fluorescence emission maximum 810 nm. Unconjugated NIRF dye (6S-ICG molecular weight ∼1700 g/mol, free carboxylic acid instead of linkage to polymer) served as control probe in the *in vivo* experiments.

### Mouse Model of Acute Allergic Asthma

Mice were sensitized intraperitoneally (i.p.) at day 0 and day 21 with 10 µg ovalbumin (OVA) dissolved in 200 µl PBS. At day 28 and day 29 mice were treated intranasal (i.n.) with a solution of 100 µg OVA/50 µl PBS/mouse. Healthy age and gender matched BALB/c mice served as controls. Histology of H&E stained lung sections was performed at 76 hrs post last challenge

### Optical Imaging Scan

48 mice were examined by optical imaging (Table 2). Mice were shaved over the lung area prior to the scanning procedure in order to reduce scattering of the signal from fur. Throughout all imaging sessions, mice were anesthetized with vaporized isoflurane at 1.8–2 volume % as described [2]. The anesthetized mice were placed inside an Optix MX2 acquisition system (Advanced Research Technologies, Montreal, Canada) and gently fixed on a heated block (37°C) for the entire duration of data acquisition.

**Table 2:** Experimental design of optical imaging biodistribution study.

	Control Dye	dPGS-NIRF		ProSense	MMPSense
Measurement time (hours)	4	4	24	24	24
**Healthy mice (number)**	5	6	5	2	2
**Asthmatic mice (number)**	5	6	10	3	4

All *in vivo* analyses were preceded by native scans of the mice prior to NIRF probe injection to provide a base line for later analysis. At 72 hrs after the last OVA challenge, mice were injected intravenously (i.v.) via the tail with 100 µl of one of the following: dPGS-NIRF (2.6 nmol, polymer/dye = 1/3), free NIRF dye (3.6 nmol), 100 µl (5 nmol) of either MMPSense (MMPSense®, Perkin Elmer) or ProSense (ProSense®, Perkin Elmer), all dissolved in 0.9% NaCl. The amount of injected dPGS-NIRF and NIRF solutions was calculated based on the weaker fluorescence signal of dye in the conjugate than in the unconjugated control dye. Exact numbers of animals in each group are shown in Table 2.

### *In vivo* and ex vivo Optical Imaging

Animals with acute asthma and wild type controls were scanned at 4 and 24 hrs post i.v. dPGS-NIRF or NIRF dye administration. For the MMPSense and ProSense, scans were performed 24 hrs after probe administration. According to the supplier (PerkinElmer), this time point constitutes the peak activation of these probes [9]. All *in vivo* data was acquired by using the small-animal time-domain Optix MX2 preclinical NIRF-imager (Advanced Research Technologies, Montreal, CA), equipped with four pulsed laser diodes and a time correlated single photon counting detector [24]. This system works in reflection mode applying a raster acquisition scheme, measuring and analyzing fluorescence response to pulsed excitation for each excitation spot by creating fluorescence photon time of flight histograms. In all imaging experiments applying the dPGS-NIRF and control dye, a 785 nm pulsed laser diode with a repetition frequency of 80 MHz was used whereas for the MMPSense and ProSense studies a 670 nm pulsed laser diode with a repetition frequency of 80 MHz was applied. Fluorescence emission was accordingly collected with an 800 nm long pass filter for dPGS-NIRF and control dye and a 700 nm long pass filter for both MMPSense and ProSense to block the excitation light. Two-dimensional regions of interest (ROIs) were selected, and laser power, integration time (repetition time of the excitation per raster point), and scan step size were optimized according to the emitted signal. Prior to probe application, mice were scanned to obtain background images. These background signal intensities recorded with the baseline image for each animal before the injection of the probe was subtracted from each post injection image. At the end of the last imaging session, 4 and 24 hrs after dPGS-NIRF/NIRF dye i.v. injection, animals were sacrificed and *ex vivo* optical imaging of the explanted lungs was performed. To calculate the total lung fluorescence intensity (Ilung) in each scan, fluorescence intensities were normalized with the laser power used for excitation and summed up in ROI's encompassing the whole organ. Lungs were then preserved in formalin for histological analysis.

### Image processing

Image analysis was done using OptiView (2.02.00), the proprietary software developed for the Optix device. All data sets of mice receiving dPGS-NIRF, free NIRF dye control as well MMPSense and ProSense were normalized for different excitation laser power and variations of the used integration time and therefore expressed in normalized counts [NC], an arbitrary unit. Average fluorescence intensity was calculated within a region of interest covering the whole lung for every sample (x) and time point (t) as 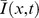 and subtracted by the base line intensity within the same region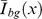. To remove the influence of different brightness of all applied probes, ratios 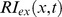 between the average intensity of the sample (x) and the mean average intensity of the control group for each experiment (ex) and time point (t) were calculated and denominated as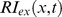.
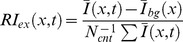
(1)



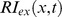
 can be interpreted as contrast or probability to distinguish asthma mice from controls at certain time points and was therefore used for comparison of the different studies and statistical calculations.

### Histological analysis of lung inflammation

Following *ex vivo* image analysis performed 76 hrs after the last ovalbumin challenge, tissue samples were fixed in 10% buffered formalin and embedded in paraffin. To evaluate allergic lung inflammation, 3 µm thick lung sections containing main stem bronchi were stained with hematoxylin and eosin (H&E). A blinded grading of the slides was done to evaluate the intensity and extent of inflammation according to our semi-quantitative scoring system. For intensity of inflammation: 0 – no inflammatory infiltrates; 1 – inflammatory infiltrates in central airways; 2 – inflammatory infiltrates extending to middle third of lung parenchyma; and 3 – inflammatory infiltrates extending to periphery of the lungs. For extent of inflammation: 0 – no inflammatory infiltrates; 1 – inflammatory infiltrates present in one third of lung surface; 2 – inflammatory infiltrates spreading up to two thirds of lung surface; 3 – inflammatory infiltrates present in more than two thirds of lung surface. Data are presented as histological score calculated as the sum of intensity and extent of inflammation for each sample. For detection of mucus-containing cells in lung tissue, adjacent 3 µm sections containing main stem bronchi from each lung specimen were stained with periodic acid-Schiff (PAS) and counter stained with hematoxylin. Slides were examined blinded for the treatment and mucus overproduction was scored as: Grade 0 – no mucus producing cells in airways; Grade 1 – few mucus producing cells in central airways; Grade 2 – mucus producing cells detected in middle airways; and Grade 3 – mucus producing cells extending to respiratory bronchioles. In borderline cases, an intermediate grade was used (0.5; 1.5 or 2.5), extending the scoring to a total of seven grades.

### Serum OVA-specific immunoglobulin

For the measurement of OVA-specific immunoglobulin (Ig) G1, ELISA plates were coated with OVA at 10 µg/ml overnight at 4°C. The plates were washed and blocked with 2% bovine serum albumin in PBS with 0.05% Tween 20 for 2 hrs at RT. Then sera were titrated onto the plates and incubated for 24 hrs at 4°C before washing. Plates were incubated for an additional 2 hrs at 4°C with biotinylated anti-IgG1 (Southern biotechnology associates Inc., Birmingham, AL, USA) detection mAb, followed by incubation with streptavidin horseradish peroxidase (Southern biotechnology) for 1 h at RT. Plates were washed and incubated with TMB substrate solution (100 µl/well, BD OptEIATM, Becton Dickinson Biosciences) for 10 min at RT. The reaction was stopped with 100 µl of 0.18 M H2SO4 and the plates were measured at 450 nm.

### In vitro analysis of serum binding

The serum binding of 6S-ICG was determined in vitro by incubation with pooled human serum (PAA) with dye concentration of 5 µg/ml [25]. The sample was placed in a Centriprep micropartition unit NWML 30 kDa (Milipore, Billerica, USA), and centrifuged at 5000 g for 20 min. The protein-bound 6S-ICG and the free dye in the ultrafiltrate was quantified spectrophotometrically (Beckman Coulter, USA).

### Fluorescence microscopy

Detection of injected dPGS-NIRF probe or unconjugated NIRF dye 6S-ICG in lungs of control and asthmatic mice was carried out by fluorescence microscopy. In order to correlate NIRF fluorescence signals from the probes to inflammatory sites, lungs were counterstained with at anti-mouse F4/80 antibody. detecting macrophages Two-micrometer-thick sections were cut from paraffin blocks, the slides were first processed for avidin/biotin and protein blocking steps using xylol and decreasing alcohol concentration for deparaffination and rehydration and later incubated with the primary antibody rat anti-mouse F4/80 (AbD Serotec, Oxford, UK), dilution factor 1∶100 at 4°C overnight. After the incubation with the primary antibody, the samples were incubated with secondary biotinylated antibody goat anti-rat (BioLegend, San Diego, USA), dilution factor 1∶200 at RT for 1 hour, and then with streptavidin- Alexa 555 (Molecular Probes, Life Technologies Corporation, USA) dilution factor 1∶400 at RT for 1 hour. DAPI was diluted in the mounting media and used as nuclear counterstaining. Fluorescence was analyzed with a Zeiss Axiovert 200 M inverted microscope (Carl Zeiss, Germany) equipped with a xenon lamp and a high sensitivity ORCA-AG digital camera (Hamamatsu, Japan). Data were acquired with AxioVs40 software (Carl Zeiss). Filter settings were as followed: DAPI: Ex: BP 365/25 (+/−12.5); FT 395; Em: BP 445/50 (+/−25); Cy7: BP 708/75 (+/−37.5); FT 757; BP 809/81 (+/−40.5); Alexa555: BP 546/12 (±6); FT 580 and LP 590 filter. Subsequent analyses were performed using the java-based image processing program ImageJ.

### Statistical Analysis

Statistical verification of the differences of 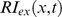 between asthmatic and control mice for each experiment and time point was done using an unpaired Welch Two Sample t-test implemented in the PAST statistic software [26]. A p-value of less than 0.05 was considered significant.

Histological scores between groups were compared using One-way ANOVA followed by Tukey's multiple comparison test. GraphPad Prism (v.5.00, GraphPad Software, San Diego, CA) was used for data analysis.
